# Influenza A Virus PB1-F2 Gene in Recent Taiwanese Isolates

**DOI:** 10.3201/eid1004.030412

**Published:** 2004-04

**Authors:** Guang-Wu Chen, Ching-Chun Yang, Kuo-Chien Tsao, Chung-Guei Huang, Li-Ang Lee, Wen-Zhi Yang, Ya-Ling Huang, Tzou-Yien Lin, Shin-Ru Shih

**Affiliations:** * National Health Research Institutes, Taipei, Taiwan; † Chang Gung University, Tao-Yuan, Taiwan; ‡ Chang Gung Memorial Hospital, Tao-Yuan, Taiwan; § Center for Disease Control and Prevention, Taipei, Taiwan; ¶ Chang Gung Children’s Hospital, Tao-Yuan, Taiwan

**Keywords:** PB1-F2, PB1 gene, influenza A virus

## Abstract

Influenza A virus contains eight RNA segments and encodes 10 viral proteins. However, an 11th protein, called PB1-F2, was found in A/Puerto Rico/8/34 (H1N1). This novel protein is translated from an alternative open reading frame (ORF) in the PB1 gene. We analyzed the PB1 gene of 42 recent influenza A isolates in Taiwan, including 24 H1N1 and 18 H3N2 strains. One H1N1 isolate and 17 H3N2 isolates contained the entire PB1-F2 ORF of 90 residues, three amino acids (aa) longer than the PB1-F2 of A/Puerto Rico/8/34 at the C terminal. The one remaining H3N2 isolate encoded a truncated PB1-F2 with 79 residues. The other 23 H1N1 isolates contained a truncated PB1-F2 of 57 aa. Phylogenetic analysis of both the HA and the PB1 genes showed that they shared similar clustering of these Taiwanese isolates, suggesting that no obvious reassortment occurred between the two genomic segments.

Influenza A virus is a prevalent pathogen with substantial pandemic potential (*1*). The influenza pandemic in 1918 was the most catastrophic in history, claiming more than 20 million lives (*2*). Influenza epidemics continue to occur every 2 to 3 years, causing substantial illness and death. Influenza A virus has eight segments of negative-stranded RNA genome and encodes 10 viral proteins (*3*). However, an 11th influenza A viral protein was recently found. This new protein product was discovered serendipitously during a systematic search of potential antigenic peptides recognized by CD8+ T lymphocytes encoded by influenza virus A/Puerto Rico/8/34 (H1N1) (*4*). Unlike other influenza A viral proteins, this novel protein has several unusual features. It is absent from some influenza virus isolates, expresses different levels among individual infected cells, degrades rapidly, and localizes to mitochondria. This protein is called PB1-F2 because it is translated from an alternative open reading frame (ORF) in the PB1 gene. The PB1 gene has an inefficient start codon, according to Kozak’s analysis (*5*), which explains why PB1-F2 can be translated when an alternative start codon is used at nucleotide positions 119 to 121 based on the PB1 gene of A/Puerto Rico/8/34 (*4*).

Since PB1-F2 induced apoptosis in a cell-specific manner and might be important in pathogenesis, do the recently circulated influenza isolates possess the alternative ORF? This study analyzed 42 influenza A isolates in Taiwan from 1995 to 2001. The sequence of the hemagglutinin gene (HA) was also analyzed for further genetic characterization of these isolates (*6*–*8*).

## Materials and Methods

### Clinical Isolates

The clinical specimens examined were throat swab specimens collected from the Clinical Virology Laboratory, Chang Gung Memorial Hospital (Contract Laboratory of the Center for Disease Control and Prevention, Taiwan). The specimens were added to Madin-Darby canin kidney (MDCK) cells. Typing of the influenza A virus then was conducted by using immunofluorescence assay (IFA) by type-specific monoclonal antibody (Dako, Cambridgeshire, UK). Moreover, subtyping was conducted by reverse transcription–polymerase chain reaction (RT-PCR) with subtype-specific primers (Table) (*9*,*10*). Virus isolates were stored at -80°C until use.

**Table:** Primers used for reverse transcription–polymerase chain reaction

Primer/nt position	Sequence	Size (bp)
H1F-78^a^	GATGCAGACACAATATGTATAGG	611
H1R-689^a^	CICTACAGAGACATAAGCATTT	
H3F-114^b^	TCAGATTGAAGTGACTAATGCT	976
H3R-1120^b^	AATTTTGATGCCTGAAACCGT	
PB1F-32^c^	TCAATCCGACCTTACTTTTC	409
PB1R-441^c^	AGCAGGCTGGTTTCTATTTA	

^a^Genomic position based on A/Puerto Rico/8/34(H1N1) nc002017. ^b^Genomic position based on A/Hong Kong/1/68(H3N2) af348176. ^c^Genomic position based on A/Puerto Rico/8/34(H1N1) isdn13420.

### RNA Extraction and RT-PCR

The clinical isolates were passed in MDCK cells, and the supernatant was used for viral RNA extraction with the Viral RNA Extraction Miniprep System kit (Viogene, Sunnyvale, CA). Viral RNA was amplified into double-stranded DNA by RT-PCR by using the Ready-To-Go RT-PCR Beads (Amersham Biosciences, Piscataway, NJ). The Table lists the primers used for RT-PCR, and the RT-PCR program in all cases was as follows: 42°C for 30 min, 95°C for 5 min, followed by 40 cycles of 95°C for 1 min, 50°C for 1 min, 72°C for 1.5 min, and a final elongation step of 72°C for 10 min. The final product was stored at 4°C.

### Nucleotide Sequence Analysis

The RT-PCR product was purified by using the QIAquick Gel Extraction Kit (Qiagen, Valencia, CA). The nucleotide sequence of the purified fragments was determined by using an automated DNA sequencer. A 561- to 564-nt HA sequence was obtained for H1N1 isolates from genomic position 120 to 683 of A/Puerto Rico/8/34 (H1N1), and an 844-nt sequence was obtained for H3N2 isolates from genomic position 187 to 1031 of A/Hong Kong/1/68 (H3N2). A 300-nt PB1 sequence was obtained from genomic position 104 to 403, covering the entire PB1-F2 gene from position 119 to position 379 of A/Puerto Rico/8/34 (H1N1). Sequence analysis, including pairwise sequence alignment and protein translation, was conducted with the Lasergene software, version 3.18 (DNASTAR, Madison, WI) (*11*). Multiple sequence alignment was conducted with Clustal W, version 1.81 (*12*), with a gap opening penalty of 15 and a gap extension penalty of 6.66. The phylogenetic analysis was performed with PHYLIP (*13*,*14*), version 3.573c, with a Kimura 2-parameter distance matrix (program dnadist) and the neighbor joining method (program neighbor). Support for tree topology was determined by bootstrap analysis with 1,000 pseudo-replicate datasets generated by the seqboot program in PHYLIP. A consensus tree was obtained by the consense program, and the topology was viewed with TreeView, version 1.6.6. Secondary structures of the sequences were predicted by using the NNPREDICT program (http://www.cmpharm.ucsf.edu/~nomi/nnpredict.html) (*15*). The reference sequences were obtained from GenBank. All human influenza A viruses with H1N1 or H3N2 subtypes whose PB1 genes could be translated into a putative PB1-F2 ORF were included for comparison with the Taiwanese strains used in this study. All human influenza A viruses with other subtypes or nonhuman influenza A viruses capable of being translated into PB1-F2 were also studied.

### Nucleotide Sequence Accession Number

The nucleotide sequence data reported in this study were deposited to the GenBank nucleotide sequence database with accession numbers AY303701 to AY303752, AF139930 to AF139940, and AF362778 to AF362820.

## Results

### Influenza A isolates in the Clinical Virology Laboratory

**Figure 1:**
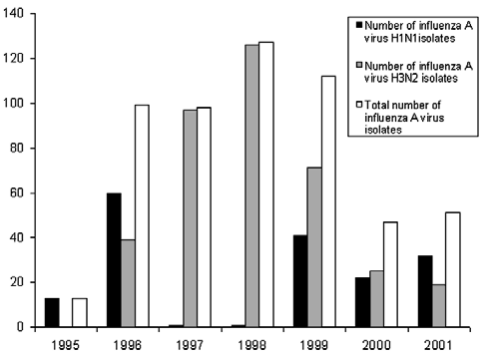
Number of isolated influenza A viruses in Clinical Virology Laboratory, Chang Gung Memorial Hospital, a contract laboratory of the Center for Disease Control and Prevention—Taiwan.

A total of 8,229 clinical specimens were received for diagnosis of the respiratory tract virus at the Clinical Virology Laboratory, Chang Gung Memorial Hospital, from 1995 to 2001. Five hundred and forty-seven influenza A viruses were isolated from these specimens, including 170 H1N1 and 377 H3N2 isolates. Notably, no H3N2 isolates were found in 1995, and only one H1N1 isolate each was found in 1997 and 1998 (Figure 1).

### PB1 Sequence Analysis

**Figure 2:**
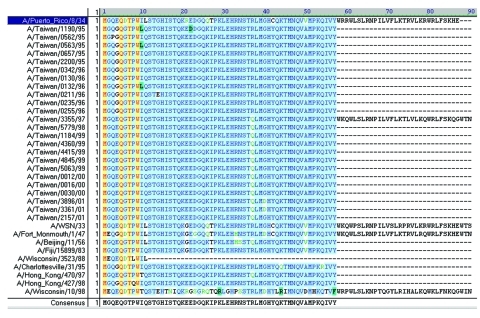
Alignment of putative PB1-F2 amino acid sequences of 24 Taiwanese H1N1 strains and 10 H1N1 reference strains. Most strains, including 23 Taiwanese strains and 5 reference strains, contained a truncated open reading frame (ORF) 57 residues long. One reference strain, A/Wisconsin/3523/88, had the ORF truncated at 11 residues. PB1-F2 of A/Puerto Rico/8/34 with 87 residues is placed on top of the alignment. One Taiwanese strain and three reference strains each contained a complete PB1-F2 of 90 residues. The program AlignX in VectorNTI Suite produced the alignment.

**Figure 3:**
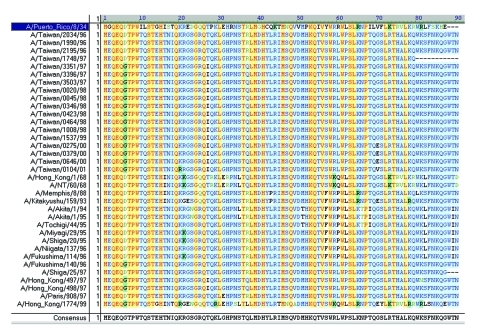
Alignment of putative PB1-F2 amino acid sequences of 18 Taiwanese H3N2 strains and 17 H3N2 reference strains. PB1-F2 of A/Puerto Rico/8/34 (H1N1) with 87 residues is laid over the alignment for reference. Most strains contained a PB1-F2 ORF 90 residues long. One Taiwanese strain, A/Taiwan/1748/97, encoded a truncated open reading frame (ORF) with 79 residues, and one reference strain, A/Shiga/25/97, encoded a 87-residue product as in A/Puerto Rico/8/34 (H1N1). The program AlignX in VectorNTI Suite produced the alignment.

The start codon for the PB1 gene is located at positions 25 to 27, while the stop codon is at positions 2296 to 2298, yielding a PB1 protein with 757 residues (*16*,*17*). The complete ORF for PB1-F2 in A/Puerto Rico/8/34 (H1N1) is from position 119 to 379, which encodes an 87-residue protein (*4*). To determine the existence of PB1-F2 in these Taiwanese influenza A isolates, 42 of them were randomly selected between 1995 and 2001, including 24 H1N1 strains and 18 H3N2 strains. Ten H1N1 and 17 H3N2 reference strains were also included for the PB1 sequence analysis. The selection was based on an extensive search for all human H1N1 and H3N2 influenza viruses in GenBank, whose PB1 nucleotide sequences were shown to alternatively translate into a PB1-F2 gene. Figures 2 and 3 show the translated PB1-F2 amino acids for H1N1 and H3N2 strains. The amino acid sequence of PB1-F2 for A/Puerto Rico/8/34 (H1N1) was included in both figures as a template. All 24 H1N1 influenza A viruses contained an alternative start codon (AUG) at positions 119 to 121 in the PB1 gene, which translated into Met (M) and marked the beginning of PB1-F2 ORF. Most of these H1N1 strains encountered a stop codon (UAG) at positions 290 to 292, resulting in the production of a 57-residue peptide and covering only a truncated PB1-F2. One isolate A/Taiwan/3355/97 (H1N1), however, covered the entire ORF (87-residue) because a non-stop codon UGG was found at equivalent positions at which other Taiwanese H1N1 encountered a stop codon. The translation continued and eventually stopped at positions 389 to 391, which encoded a putative protein of 90 residues, three residues longer than the PB1-F2 protein in A/Puerto Rico/8/34. For the nine reference strains in addition to A/Puerto Rico/8/34, three strains (A/WSN/33, A/Fort Monmouth/1/47 and A/Wisconsin/10/98) encoded a PB1-F2 of 90 residues, five strains (A/Beijing/11/56, A/Fuji/15899/83, A/Charlottesville/31/95, A/Hong Kong/470/97, and A/Hong Kong/427/98) encoded a truncated ORF of 57 residues, and one strain (A/Wisconsin/3523/88) encountered an exceptionally early stop codon and ended in only 11 residues. Most of the H3N2 isolates analyzed in this study, on the other hand, covered the full-length of PB1-F2 ORF of 90 residues as found in A/Taiwan/3355/97 (H1N1). Only two exceptions were observed, including one Taiwanese strain A/Taiwan/1748/97 with a truncated PB1-F2 ORF of 79 residues, and one reference strain A/Shiga/25/97 with 87 residues as in A/Puerto Rico/8/34 (H1N1).

**Figure 4:**
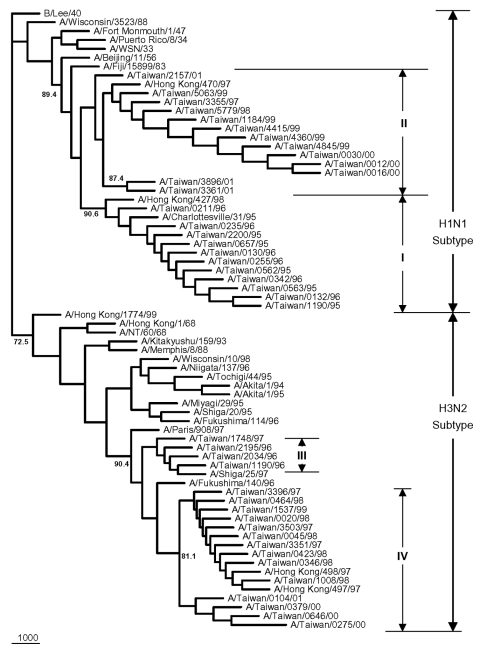
Phylogenetic tree of influenza A viruses for their PB1 gene nucleotide sequences. Apart from the 42 Taiwanese isolates obtained in this study, 27 reference strains were included; these were selected on the basis of an extensive search of all human H1N1 and H3N2 influenza viruses from GenBank, whose PB1 sequences were shown to be able to be translated into the putative PB1-F2 gene. The tree was rooted with B/Lee/40. All strains were separated into two groups according to their subtypes. Sequence analysis was conducted using the software Lasergene, Clustal W and PHYLIP with 1,000 replicates. All sequences are 300-nt long from genomic position 104 to 403 based on the PB1 gene of A/Puerto Rico/8/34, which covers at least the entire PB1-F2.

The nucleotide sequence identities for PB1 gene were 96.3%–100% among the 24 Taiwanese H1N1 strains, and 97.3%–100% among the 18 H3N2 strains, based on a 300-nt segment that covers the entire putative PB1-F2 of interest. Figure 4 presents the PB1 phylogenetic tree for all 42 Taiwanese isolates and 27 previously selected reference strains. All Taiwanese strains were grouped into their respective subtype. The Taiwanese H1N1 strains were divided into two clusters. One contained eleven 1995–1996 isolates, which were similar to A/Charlottesville/31/95 and A/Hong Kong/427/98 (97.0%–98.6%), and the other contained 13 1997–2001 isolates, which were similar to A/Hong Kong/470/97 (99.0%–99.6%). The Taiwanese H3N2 strains were also divided into two clusters, including four 1996–1997 isolates similar to A/Shiga/25/97 (98.6%– 99.6%) and fourteen 1997–2001 isolates similar to A/Hong Kong/497/97 and A/Hong Kong/498/97 (98.3%–100.0%).

The putative PB1-F2 protein of A/Taiwan/3355/97 (H1N1) was 81.1% identical to A/Puerto Rico/8/34 (H1N1). In addition to the three extra amino acids (Trp88-Thr89-Asn90) at the C-terminal end, A/Taiwan/3355/97 (H1N1) had 14 aa that differed from the 87-residue peptide of A/Puerto Rico/8/34. Notably, two Arg residues, Arg75 and Arg79, which have a propensity to form an amphipathic helix and may be required for membrane translocation (*4*,*15*), had been changed to Leu75 and Gla79 in A/Taiwan/3355/97 (H1N1). However, A/Taiwan/3351/97 (H3N2) (Figure 3), a strain that also contains a 90-residue PB1-F2 and was isolated at approximately the same time as A/Taiwan/3355/97 (H1N1), was only 50.0% identical to A/Puerto Rico/8/34 and differed in 42 aa other than the three trailing residues. The difference between A/Taiwan/3355/97 (H1N1) and A/Taiwan/3351/97 (H3N2), both with a 90-residue PB1-F2, was 37 residues; these two strains share only 58.8% identity.

### HA Nucleotide Sequence Analysis

**Figure 5:**
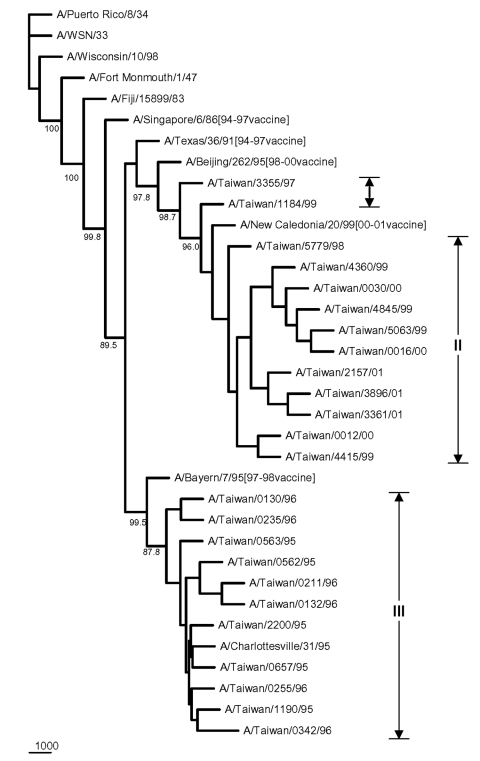
Phylogenetic tree of H1N1 influenza A viruses for HA nucleotide sequences. Five recent vaccine strains were included along with the 24 Taiwanese strains and six reference strains considered previously in Figure 4. The tree was rooted with A/Puerto Rico/8/34. Sequence analysis was conducted using the software Lasergene, Clustal W, and PHYLIP with 1,000 replicates. Sequences contained 561 to 564 nt bases from positions 120 to 683, based on A/Puerto Rico/8/34.

**Figure 6:**
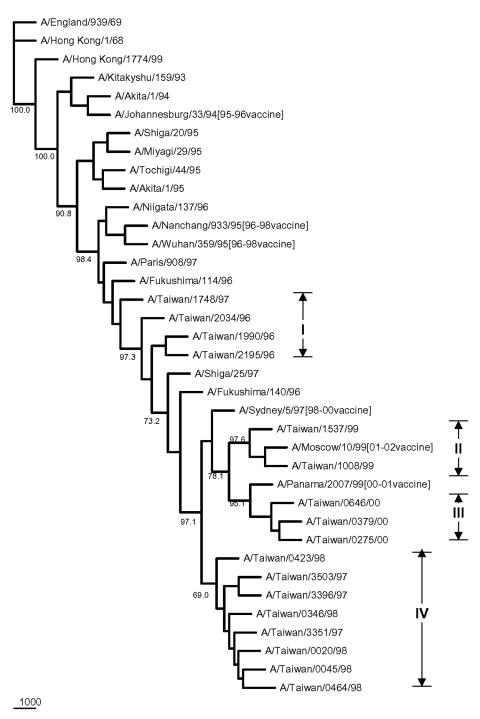
Phylogenetic tree of influenza A H3N2 viruses for HA nucleotide sequences. Six recent vaccine strains were included along with the 17 Taiwanese strains and 13 reference strains shown in Figure 4. The tree was rooted with A/England/939/69. Sequence analysis was conducted by using the software Lasergene, Clustal W, and PHYLIP with 1,000 replicates. All sequences are 844-nt long from positions 187 to 1031, based on A/Hong Kong/1/68.

The HA sequences were also analyzed to elucidate the genetic characteristics of these Taiwanese influenza A isolates. Figure 5 shows the phylogenetic tree of HA for 35 H1N1 strains, including 24 Taiwanese isolates, 6 reference strains used in earlier PB1-F2 analysis, whose HA sequences were available in GenBank, and 5 recent H1N1 vaccine strains. The sequence identity among these 24 isolates was 91.8%–100%, based on a 561- to 564-nt HA segment. Eleven 1998–2001 isolates were clustered with the 2000-2001 vaccine strain A/New Caledonia/20/99 (*18*–*20*), exhibiting a nucleotide sequence identity from 97.5% to 99.2%. Another eleven 1995-1996 isolates were clustered with A/Bayern/7/95 (*21*–*24*), the vaccine strain used in 1997–1998, and A/Charlottesville/31/95, exhibiting a nucleotide sequence identity from 97.8% to 100%. The other two Taiwanese isolates, A/Taiwan/3355/97 and A/Taiwan/1184/99, were separated from the 1998–2001 Taiwanese isolates with good bootstrap values. A/Taiwan/1184/99 was similar to A/New Caledonia/20/99, exhibiting an identity of 99.2%. A/Taiwan/3355/97, although further apart, was also similar to A/New Caledonia/20/99 too with a 97.5% identity. A/Taiwan/3355/97 was also adjacent to the preceding vaccine strain A/Beijing/262/95, as determined from phylogeny and shared a 96.4% identity. Nevertheless, all 13 Taiwanese H1N1 isolates from 1997 to 2001 exhibited a characteristic deletion mutation Lys (K) at aa 134 of the HA gene, as has been previously reported (*25*). The distinct genetic feature of possessing the entire PB1-F2 ORF in A/Taiwan/3355/97 might have separated this strain from other 23 Taiwanese H1N1 isolates in the phylogenetic tree. Figure 6 shows the phylogenetic tree of 37 H3N2 strains, including 17 Taiwanese isolates, 13 reference strains used in preceding PB1 analysis, whose HA sequences were available, 6 recent H3N2 vaccine strains, and 1 English strain as an outgroup. The nucleotide sequence identity in terms of HA gene among the 17 analyzed Taiwanese H3N2 isolates from 1996 to 2001 was 96.2%–100%, based on an 844-nt HA segment, which is higher than that among the 24 Taiwanese H1N1 isolates (91.8%–100%) under investigation. The two 1999 isolates were clustered with A/Moscow/10/99 (99.6%–99.7%), which was the vaccine strain used in 2001 to 2002 (*26*). The three 2000 isolates were clustered with the 2000–2001 vaccine strain A/Panama/2007/99 (98.8%–99.4%) (*18*). The eight 1997–1998 isolates formed their own cluster, despite a relatively low bootstrap support, and were similar to the 1998–2000 vaccine strain A/Sydney/5/97 (98.8%–99.4%) (*24*,*27*,*28*). Four other Taiwanese 1996–1997 isolates were not grouped into any of these Taiwanese H3N2 clusters. They were similar to A/Shiga/25/97 and the two 1996 Fukushima strains (97.5%–99.5%).

## Discussion

One isolate among the 24 Taiwanese H1N1 strains from 1996 to 2001 possessed the full-length PB1-F2 ORF. Most of the H1N1 isolates contained a shorter 57-residue putative PB1-F2, encountering a premature stop codon at positions 290 to 292. All of the Taiwanese H3N2 isolates, however, contained the full-length ORF from 1995 to 2001, except for A/Taiwan/1748/97, which encoded a truncated PB1-F2 of 79 residues. The putative full-length PB1-F2 of these Taiwanese strains contained 90 aa, which was three residues longer than A/Puerto Rico/8/34 (H1N1).

PB1-F2 of A/Puerto Rico/8/34 (H1N1) has been shown to localize to the mitochondria and induce cell death. According to the NNPREDICT algorithm, PB1-F2 of A/Puerto Rico/8/34 (H1N1) tends to form an amphipathic helix, extending from Leu69 to Phe83 (*4*,*15*). The predicted helix includes five basic residues. This feature enables PB1-F2 to translocate through the membrane, target the mitochondria, and trigger host cell apoptosis (*29*). The putative 90-residue-long PB1-F2 protein of A/Taiwan/3355 (H1N1) and A/Taiwan/3351 (H3N2) also possessed a predicted helix from Leu72 to Phe83. However, A/Taiwan/3355/97 (H1N1) contained three basic residues, and A/Taiwan/3351/97 (H3N2) contained four. The truncated PB1-F2 (57-residue ORF of most Taiwanese H1N1 strains or the 79-residue ORF of A/Taiwan/1748/97 [H3N2]) did not have the predicted helix. The change in the number of basic residues in those Taiwanese strains did not alter the result from NNPREDICT, that is, all full-length PF1-F2 amino acid sequences (87- or 90-residue ones) were predicted to contain a helix around the same location. Whether the reduction in the number of basic residues in the predicted helix, or the significant difference between the PF1-F2 amino acids of the Taiwanese strains and of A/Puerto Rico/8/34 (H1N1), supports functions that differ from those of A/Puerto Rico/8/34 (H1N1), remains to be investigated.

The truncated PB1-F2 (with <87 or 90 aa residues) did not contain the mitochondrial translocation signal at the C-terminal end and may lose the PF1-F2 functions described above. The earliest observation for such truncation of human H1N1 or H3N2 influenza A viruses was associated with A/Beijing/11/56 (H1N1), which has been seen in most Taiwanese H1N1 strains and four other reference strains (Figure 3), as well as in A/Taiwan/1748/98 (H3N2) (Figure 4).

All of the H1N1 Taiwanese isolates from 1997 to 2001 exhibited a stable genetic characteristic—a deletion mutation at aa 134 in the HA gene. The only H1N1 isolate in 1997, A/Taiwan/3355/97, was found to encode the entire 90-residue PB1-F2 ORF, which is 3 aa longer than the PB1-F2 of A/Puerto Rico/8/34 (H1N1). Although A/Taiwan/3355/97 (H1N1) was found to be phylogenetically related to those isolates that encode partial PB1-F2 from 1998 to 2001 in both HA and PB1 genes, this genetic marker that putatively encodes a full-length PB1-F2 was not retained in subsequent isolates. This finding suggests that the existence of a putative full-length PB1-F2 ORF in the PB1 gene might have been a disadvantage to the growth of H1N1 virus in the infected host population. However, almost all of the H3N2 isolates analyzed in this study encoded a full-length ORF of PB1-F2, including the Taiwanese strains and the reference strains from GenBank. The only exception was A/Taiwan/1748/97, which encoded a truncated PF1-F2 with 79 residues.

The 336 influenza A PB1 sequences of all species from GenBank, including all the Taiwanese and reference strains analyzed in this work, were collected to provide an overall picture of how a putative PB1-F2 can be alternatively translated from its PB1 gene. All 336 PB1 sequences examined contained the entire RNA segment equivalent to the position of PB1-F2 ORF as in A/Puerto Rico/8/34 (H1N1). Only two avian strains without a start codon were observed (A/Quail/Hong Kong/AF157/92 [H9N2] and A/duck/NC/91347/01 [H1N2]), leaving no PB1-F2 encoded. Ninety-nine human influenza A viruses were observed, among which 64 encoded a 90-residue PB1-F2, two encoded an 87-residue ORF (A/Puerto Rico/8/34 [H1N1] and A/Shiga/25/97 [H3N2] stated early section), and 33 encoded a truncated ORF (1 strain with 79 residues, 30 with 57, and 2 with 11). Of the remaining 235 nonhuman influenza A viruses, 191 encoded a 90-residue PB1-F2, 7 encoded an 87-residue ORF, and 37 encoded a truncated ORF. (Twenty-three strains had 79 residues, 2 had 63; 5 had 57; 1 had 34, and 6 had 11.) Of all the 334 translated PB1-F2, 264 (79.0%) contained a complete ORF of 87 or 90 residues.

The complete PB1 sequences of 38 influenza A viruses, including 26 human and 12 nonhuman strains, were gathered from GenBank to evaluate the genetic variation of the PB1 gene and the putative PB1-F2 gene. Each sequence is 2,271 to 2,274-bp long, covering the entire coding region of PB1 and containing a putative PB1-F2 ORF of at least 87 residues. The pairwise identities of these 38 PB1 coding sequences were from 81.2% to 99.9% for nucleotides, and 94.3%–99.7% for the translated amino acids. For the alternatively translated PB1-F2 ORF that is 261- to 270-bp long, or from nucleotide position 95 to 364 with respect to the full coding region of PB1, the pairwise identities were 79.2%–99.6% for nucleotides, and 52.2%–98.8% for the translated amino acids. In terms of the partial sequences in the forms of their regularly translated PB1 segments (from position 94 to 363, or a 70-residue peptide) equivalent to a genomic range in which the PB1-F2 is translated, the pairwise identities for these 38 strains were 92.2%–100.0%. The variation among the sequences was limited to approximately 20% in nucleotides and <8% in amino acids for the complete PB1 coding region, as well as for a partial PB1 segment (position 94 to 363) corresponding to the PB1-F2 location. The alternatively translated PB1-F2 to the equivalent piece of RNA (position 95 to 364), however, exhibited a large variation up to 50%. The two translations differed by only 1 nt. Although certain single nucleotide mutations, which might have occurred at the third position of a codon in the original PB1 translation, did not seem to substantially alter the resulting amino acid sequences, a frame shift of alternatively translating the RNA into a putative PB1-F2 apparently introduced a substantial change in the genetic heterogeneity up to 47.8%. A single nucleotide change can also easily introduce a stop codon under this new translation, which might account for the observed truncated PB1-F2 genes of various lengths. PB1-F2 protein is not essential to influenza viral replication, so this novel gene may be subject to less selection pressure than PB1 or HA during viral evolution.

Phylogenic inference drawn from PB1 sequences showed two distinct clusters within the H1N1 and H3N2 groups of the analyzed Taiwanese strains (Figure 4). Twenty-four Taiwanese H1N1 strains were grouped into cluster I (which contained eleven 1995–1996 strains) and cluster II (which contained thirteen 1997–2001 strains). This same clustering pattern was seen in the HA phylogenetic tree for H1N1 strains in Figure 5, in which the eleven 1995–1996 strains joined cluster III, and the thirteen 1997–2001 strains were in cluster I+II. A similar clustering pattern of 18 Taiwanese H3N2 strains was also observed between Figures 4 and 6. Phylogenetic analysis of both PB1 and HA genes showed no evidence of reassortment for the two viral segments among these Taiwanese isolates.

Apoptosis is an important pathogenic mechanism in influenza A virus infection. Differential induction of apoptosis was caused in the infected cells by different influenza virus strains (*30*). PB1-F2 is a mitochondrial protein that induces apoptosis. PB1-F2 was absent from certain influenza A isolates, so further investigating the correlation between the existence of PB1-F2 and the apoptosis level of influenza A virus–infected cells would be of interest.
